# A method for statistically comparing spatial distribution maps

**DOI:** 10.1186/1476-072X-8-7

**Published:** 2009-01-30

**Authors:** Rebecca S Levine, Krista L Yorita, Matthew C Walsh, Mary G Reynolds

**Affiliations:** 1 CDC/ORISE Poxvirus Program Research Fellow, Centers for Disease Control and Prevention, 1600 Clifton Road MS G-06, Atlanta, GA 30333, USA; 2 CDC/ORISE Office of the Director Research Fellow, Centers for Disease Control and Prevention, 1600 Clifton Road MS A-30, Atlanta, GA 30333, USA; 3 CDC/CCID/DVRD/Poxvirus Program Epidemiologist, Centers for Disease Control and Prevention, 1600 Clifton Road MS G-06, Atlanta, GA 30333, USA

## Abstract

**Background:**

Ecological niche modeling is a method for estimation of species distributions based on certain ecological parameters. Thus far, empirical determination of significant differences between independently generated distribution maps for a single species (maps which are created through equivalent processes, but with different ecological input parameters), has been challenging.

**Results:**

We describe a method for comparing model outcomes, which allows a statistical evaluation of whether the strength of prediction and breadth of predicted areas is measurably different between projected distributions. To create ecological niche models for statistical comparison, we utilized GARP (Genetic Algorithm for Rule-Set Production) software to generate ecological niche models of human monkeypox in Africa. We created several models, keeping constant the case location input records for each model but varying the ecological input data. In order to assess the relative importance of each ecological parameter included in the development of the individual predicted distributions, we performed pixel-to-pixel comparisons between model outcomes and calculated the mean difference in pixel scores. We used a two sample Student's t-test, (assuming as null hypothesis that both maps were identical to each other regardless of which input parameters were used) to examine whether the mean difference in corresponding pixel scores from one map to another was greater than would be expected by chance alone. We also utilized weighted kappa statistics, frequency distributions, and percent difference to look at the disparities in pixel scores. Multiple independent statistical tests indicated precipitation as the single most important independent ecological parameter in the niche model for human monkeypox disease.

**Conclusion:**

In addition to improving our understanding of the natural factors influencing the distribution of human monkeypox disease, such pixel-to-pixel comparison tests afford users the ability to empirically distinguish the significance of each of the diverse environmental parameters included in the modeling process. This method will be particularly useful in situations where the outcomes (maps) appear similar upon visual inspection (as are generated with other modeling programs such as MAXENT), as it allows an investigator the capacity to explore subtle differences among ecological parameters and to demonstrate the individual importance of these factors within an overall model.

## Background

Ecological niche modeling is an emerging spatial mapping technology designed to characterize and map the ecological niche distributions occupied by species [1]. A challenge in the field of such modeling has been determining whether independent distribution maps for a single species – maps generated through similar processes, but with distinct sets of ecological inputs – are significantly different from one another. Here, we describe a rigorous method for comparison of model outcomes, which allows evaluation of whether the range and intensity of prediction is significantly different between projected distributions.

We performed an ecological niche modeling study to describe the distribution of monkeypox disease in humans throughout Africa, its only endemic region [2]. Monkeypox disease is caused by a virus of the same name that produces a serious, smallpox-like illness in humans. Little specific ecological information is known about monkeypox virus; neither the complete geographic distribution, natural reservoir and/or intermediate zoonotic host(s), nor the principal route of transmission between animals and humans and between humans is fully understood [3]. Our goal in creating a spatial model of human disease occurrence was to describe which ecological conditions might be most significant in determining disease distribution [4].

For our purposes, we utilized the software GARP (Genetic Algorithm for Rule-set Production), which models ecological niches of species and predicts their distributions in geographic space [5]. GARP is available through the world-wide web free of charge at . Species distributions are obtained with GARP using a unique genetic algorithm that creates a series of rules relating specific ecological characteristics to known species occurrences. The user input is twofold: a set of ecological parameters and a set of species occurrence points. The outcome is a predictive spatial distribution map of the species' overall ecological niche based on the input factors which most closely associate environmental characteristics and a species' presence or absence in a geographic region [1,5].

The GARP modeling algorithm itself incorporates stringent internal accuracy tests for evaluating the validity of predicted distribution models. Internal model validation occurs through both iterative solving for solution optimization as well as division of input data into multiple training and testing sets for independent confirmation. Distribution output at the conclusion of a modeling session is accompanied by a table of statistics assessing significance, including a chi-square test and resulting *p*-value for each generated map [5]. Several studies have also successfully validated the accuracy of individual spatial distribution models produced via rigorous external test methods, such as using independent data sets or additional field sampling of selected species [6-9].

Although internal tests for model accuracy are available within the framework of the GARP algorithm, a limitation of this and many other spatial modeling technologies is a failure to address the endpoint requirement of being able to compare, in a statistically rigorous fashion, the degree to which individually-generated model results agree with one another. For this example, we wished to determine whether a predicted distribution for human monkeypox disease produced from ecological layers *a*, *b*, and *c *was statistically different from a predicted distribution for human monkeypox disease produced from ecological layers *a *and *b *alone. The following presents a method for performing a statistically based comparison of such spatial maps of the same size and scale. We embarked on this project for a specific purpose, namely we needed to determine which ecological parameters were the most significant in determining the available niche for human monkeypox disease. While the method presented herein stemmed from our need to compare variously derived ecological niche models to one another, the procedure is not specific to that purpose alone and constitutes a potentially valuable contribution to the field of spatial mapping as it can be used to compare any two (or more) distribution maps of equivalent dimensions, such as those generated through the use of other modeling applications, e.g., MAXENT [10].

## Results

**Figure 1:**
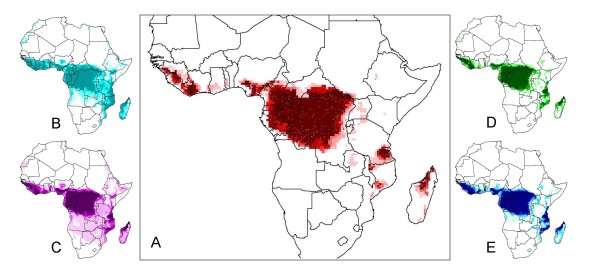
**Spatial results from 'jackknife procedure' to determine relative significance of various ecological parameters**. Figure 1A shows the comprehensive map made with all parameters – each jackknife map was compared for similarity via statistical analysis with this comprehensive map. Figures 1B-E show jackknife maps created each with the exclusion of one parameter. Excluded parameters shown are: (B) Precipitation, (C) Flow Direction, (D) Land Cover, and (E) Frost Days. These four maps were selected for visualization based on statistical results of the jackknife test shown in table 1 [2].

Previously, we used ecological niche modeling software to develop a predicted geographical distribution of human monkeypox disease, shown in figure 1A[2]. Thirteen environmental parameters (N = number of parameters) were selected for inclusion in this comprehensive ecological niche model and are listed in table 1[2]. In order to determine the relative importance of each of the ecological parameters used for the predictive distribution of human monkeypox disease, a 'jackknife procedure' was performed as outlined by Peterson and Cohoon [11]. The jackknife process (which is not unique to GARP) involved the construction of a series of individual maps (each map being derived from summing 10 high-quality individual models), with each model-derived map missing one of the layers that was used to create the comprehensive map. This resulted in N-1 predicted distribution maps of identical size to each other and to the comprehensive map, where a jackknifed map represented the range of the disease *without *that particular parameter considered. A selection of these jackknifed maps is shown in figures 1B–1E.

**Table 1:** Summary of statistical analysis of 'jackknife procedure' used to determine environmental importance of ecological parameters (re-printed with permission from [2]).

**N-1 Excluded Parameter**	**Difference from Comprehensive Map**					
	
	*Mean*	*Std Dev*	*t Value*	*Pr > |t|*	*%*	*Kappa*
Aspect	0.227	1.1201	75.25	< .0001	14.90913	0.8471

Diurnal Temp Range	-0.162	1.462	-41.09	< .0001	16.00786	0.8074

Elevation	-0.266	1.265	-78.19	< .0001	14.59951	0.8366

Flow Accumulation	-0.014	0.9739	-5.36	< .0001	12.44882	0.8683

Flow Direction	-0.362*	1.0288	-130.6*	< .0001	11.30027	0.818

Frost Days	-0.005	1.0807	-1.62	***0.1063§***	13.99543	0.8562

Land Cover	0.3947*	1.1047	132.67*	< .0001	15.73201	0.8418

Precipitation	-1.484*	2.1541*	-255.8*	< .0001	26.04734*	***0.6299±***

Minimum Temp	0.2259	1.0138	82.75	< .0001	13.24499	0.8606

Mean Temp	-0.204	1.1033	-68.74	< .0001	13.09959	0.8392

Maximum Temp	-0.298	1.4251	-77.65	< .0001	13.2078	0.8063

Topographic Index	-0.026	0.957	-9.94	< .0001	12.70847	0.8627

Wet Days	-0.134	1.2503	-39.82	< .0001	14.66416	0.833

(*) Indicates extreme values of mean difference, standard deviation, t value, and % difference. (§ Indicates the p-value for which exclusion of this parameter from the model caused no significant difference. (±) Indicates the kappa value for which exclusion caused overall model agreement to drop below significance, indicating the model loses internal accuracy without inclusion of this parameter.

In order to assess the relative importance of each ecological parameter in the development of the model, it was necessary to statistically compare each of the individual jackknife maps to the comprehensive map. This analysis goes one step beyond the scope of previously existing technology. All maps were created initially as ArcInfo grids; however, we used a free downloadable Avenue script to export the ArcInfo grids as ascii raster grids, or numerical representations of images where each pixel is represented by a unique cell with a specific score for each parameter . Possible scores for each pixel ranged from 0–10, with 0 indicating that the disease's ecological niche was absent from that pixel and 10 indicating the maximum positive prediction for presence. Of key importance is that the maps were of identical dimensions and classified using an equivalent continuous scale, meaning that each raster grid had the same number of cells and possible scores.

ESRI's *Arc View GIS version 3.3 *for windows, plus the *Spatial Analyst *extension Geographic Information Systems (GIS) software was utilized for creation and manipulation of spatial maps. Statistical analyses were performed using the *SAS System for Windows, Version 9 *.

### Step 1 – data formatting

Upon transforming each GARP-generated dataset as a rasterized ascii file with rows and columns corresponding to the map's coordinates, each map was exported as a grid of identical size containing 886 rows and 739 columns. Next, we created a one-dimensional array with the number of positions equal to the number of x-coordinates in the map, thereby transforming the file from its original grid format to a single column of data. The resulting dataset contained one observation per cell and preserved both the unique score and position of each pixel. This subsequent ascii transformation array created using SAS corresponded to a dataset containing 654,754 unique observations (rows) that equaled the number of pixels in the original coverage area. This process was repeated for each spatial distribution map we wished to compare (12 jackknife maps plus 1 comprehensive map).

The individual array datasets from each map were merged together to form a single dataset with one column per jackknife map, and one row per unique pixel position. The variables for each observation consisted of the pixel identifier (its row) and the pixel score (0–10) from each column. To increase computational efficiency, we deleted all observations (i.e. rows) which had pixel scores of zero for all maps before beginning statistical analysis. These 100% niche-absence pixels represent areas that would never include predicted niche for this "species," such as oceans or desert regions. This data culling limited the data only to those pixels having at least one non-zero score thereby providing meaningful comparisons and facilitating statistical calculations. After deleting all observations for which the score was zero for all jackknife maps, 137,857 pixels remained which had at least one non-zero score.

### Step 2 – variable creation

To complete the dataset to be used in analysis for pixel-by-pixel comparison between each individual jackknife map and the comprehensive map, we created new variables to represent the difference in pixel scores between each pair of maps. Consider a particular pixel *y *whose score in jackknife layer *a *was 7 and score in the comprehensive map *b *was 5. The pixel difference (d_n_) for pixel *y *was represented by the difference in scores, in other words d_y _= 2. A mean difference in pixel score of zero would satisfy the null hypothesis (if d_n _= 0 pixel scores are the same) i.e., that such a jackknife map was identical to the comprehensive map, thus demonstrating that the missing layer had little to no influence on the predicted niche distributions.

### Step 3 – statistical testing

As the sample population of map pixels was quite large (137,857 pixels), mean pixel difference scores were assumed to have a normal distribution. Therefore, a two sample Student's t-test was used to evaluate the null hypothesis and generate statistics including: mean difference, standard deviation, *t*-value, and *p*-value for the t-test. Results showing the mean difference, standard deviation, t-value, and *p*-value for the t-test of each ecological parameter are shown in table 1[2]. Based on this preliminary analysis, we determined that when removed from the model, precipitation, flow direction (water), and land cover most profoundly altered the predicted distribution of human monkeypox disease, suggesting their importance in determining the range for this model. Removal of precipitation and flow direction caused pronounced commission of the range while removal of land cover caused a relative omission (shown by highest differences from mean pixel scores and disproportionately extreme t-values). Furthermore, when removed, precipitation caused the mean standard deviation of the model to increase nearly two-fold, to 2.15 as compared to the average 1.15. All parameters were found statistically significant for model inclusion (*p *< 0.0001) with the exception of frost days (*p *= 0.11), indicating that frost days did not significantly contribute to this distribution model of human monkeypox disease.

The use of t-tests and other independent two-group tests evaluated only whether the mean pixel score was the same in both groups, whereas we were also concerned with the distribution of scores. In order to compare the relative difference of each map, we first utilized weighted kappa statistics using the FREQ procedure in SAS. Kappa statistics are most often used to evaluate inter-rater reliability when judging a common stimulus. In the case of map comparison, the 'raters' were the maps being compared, while the stimulus was the data provided by the variables (each map being compared) and the agreement objective was the pixel score generated by each map. These statistics were weighted based on the Cicchetti-Allison method so as to consider deviations further from the mean as more divergent than deviations closer to the mean. A kappa value of 1 indicates perfect agreement between raters and a value of 0 indicates no more agreement than that expected by chance. Weighted kappa values between 0.8 and 1 are generally accepted as having excellent agreement between the raters; values falling below 0.8 may be considered less statistically significant [12]. We found that precipitation, when excluded from the overall ecological niche model of human monkeypox disease, was the sole layer causing the kappa coefficient to drop below significance (0.63), as shown in table 1[2].

**Figure 2:**
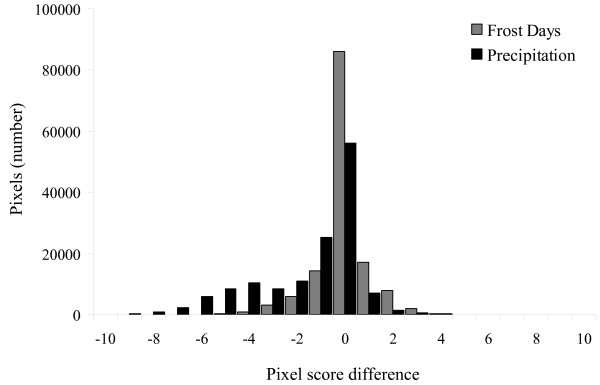
**Histogram showing the distribution of pixel score differences between the comprehensive map and two jackknife maps**. Exemplar jackknife maps produced by exclusion of 'mean annual precipitation' and 'frost days' layers from the model were chosen to illustrate distributions resulting in significant versus non-significant findings, respectively. The sign of the mean difference (positive or negative integer value) indicates whether each jackknife layer map over-predicted (negative) or under-predicted (positive) the distribution as compared to the comprehensive map. A jackknife parameter having many pixels with values of or close to zero (difference = 0) presents a distribution most similar to the comprehensive map, indicating that its exclusion does not seriously affect the distribution of disease.

The second method for comparing the relative difference of each map was the creation of histograms showing the frequency distribution of pixel score differences for each ecological parameter's jackknife map as compared to the scores generated by the comprehensive map. We generated a 'score' variable by multiplying the number of pixels with a certain difference score by that value (i.e., if d_n _= 5 for 50 pixels in a jackknife map as compared to the complete map, the score would be 50*5 = 250). For negative differences (indicating over-prediction of distribution), we multiplied by negative one to get a positive score. If the summed score results for each jackknife map had many pixels with scores either the same or very close to the same as the complete distribution map, the mean difference score was closer to zero, becoming larger with a greater dissimilarity. Exclusion of precipitation and flow direction again yielded the highest divergence from the comprehensive map. The distributions of pixel difference scores for 2 exemplar jackknife maps as compared to the comprehensive map are shown in Figure 2.

Finally, we examined the frequency distribution of pixel score differences. Using absolute difference scores, we calculated the percent of pixel difference scores falling outside of one standard deviation of the mean difference in pixel score. Though the exclusion of the flow direction parameter failed to stand out, the exclusion of the precipitation parameter caused the percent difference between the jackknifed map and the comprehensive map to increase nearly two-fold (26% difference as compared with an average 14% difference). This result is shown in table 1 as percent difference [2].

The observation that multiple independent statistical tests demonstrated a significant loss of internal consistency for the overall model when precipitation was left out, strongly supported the idea that precipitation was the single most important independent ecological parameter in the niche model for human monkeypox disease.

## Discussion

It is broadly accepted that the determination of range limits for species often have at their core the effect of various ecological parameters [13,14]. Ecological niche modeling is itself a technique designed to account for multi-dimensional aspects of a species' particular habitat in ecological space and considered superior to many other first-order modeling systems precisely because it addresses many factors simultaneously in both spaces. However, we found that after a final model was created to give a description of *where *human monkeypox disease might be found; missing from the current GARP modeling system was the power to explain *why*. In other words, we lacked a method to assist in distinguishing the empirical significance of each of the diverse ecological parameters considered, thereby hindering our ability to explain why the disease was where it was and wasn't where it wasn't.

GARP models are sensitive to ecological inputs – we expect that most layers will have a meaningful (significant) contribution to the outcome. Furthermore, we expect to see significant differences when a layer is removed, otherwise the ecological inputs were selected poorly. We are most interested in this statistical method for its capacity to explore subtle differences among the ecological parameters, and feel its greatest utility is in revealing the extremes of the individual environmental factors' importance within the overall niche model. For example, information relating to frost in our model of human monkeypox disease showed that it wasn't a particularly useful parameter whereas the statistics relating to precipitation showed that it was of significant importance. In this study, the differences between the jackknifed models are not subtle, but are nevertheless in line with our expectations. We anticipate that this method will prove most useful for making meaningful comparisons when distribution maps appear similar upon visual inspection.

## Conclusion

The method described herein presents a procedure for evaluating the statistical significance of ecological parameters involved in niche modeling. Here we have applied the procedure to output created using the GARP system, but this method is broadly applicable to other spatial modeling technologies as well, such as MAXENT, which others have found to be superior to GARP [10]. While our method elucidated precipitation as a highly significant determinant in the distribution of human monkeypox disease in Africa, it is still a clear beginning step and not without limitations. Areas of improvement include an ability to employ more powerful statistical tests beyond a Student's t-test or the relatively weak kappa test, and to compare maps that are not necessarily of the same size or scale. Nevertheless, this method fills a gap in the practical application in both the fields of spatial mapping and statistics and will serve as a stepping stone for future comparative studies.

## Competing interests

The authors declare that they have no competing interests.

## Authors' contributions

RL and MG conceived of the study, participated in its coordination and execution, and drafted the manuscript. KY created the variables and performed the statistical testing. MW formatted the data. All authors read and approved the final manuscript.
